# APOC1 predicts a worse prognosis for esophageal squamous cell carcinoma and is associated with tumor immune infiltration during tumorigenesis

**DOI:** 10.3389/pore.2023.1610976

**Published:** 2023-03-08

**Authors:** Xiying Cao, Bingqun Wu, Shaoming Guo, Weixiang Zhong, Shenyu Zhu, Zuxiong Zhang, Liang Gu, Hui Li

**Affiliations:** ^1^ Department of Thoracic Surgery, Beijing Chao-Yang Hospital, Capital Medical University, Beijing, China; ^2^ Department of Thoracic Surgery, The First Affiliated Hospital of Gannan Medical University, Ganzhou, Jiangxi, China; ^3^ Department of Thoracic Surgery, Huaxin Hospital, First Hospital of Tsinghua University Beijing, Beijing, China

**Keywords:** bioinformatics, prognosis, expression, APOC1, ESCA

## Abstract

**Background:** Esophageal carcinoma (ESCA), a common malignant tumor of the digestive tract with insidious onset, is a serious threat to human health. Despite multiple treatment modalities for patients with ESCA, the overall prognosis remains poor. Apolipoprotein C1 (APOC1) is involved in tumorigenesis as an inflammation-related molecule, and its role in esophageal cancer is still unknown.

**Methods:** We downloaded documents and clinical data using The Cancer Genome Atlas (TCGA)and Gene Expression Omnibus (GEO) databases. We also conducted bioinformatics studies on the diagnostic value, prognostic value, and correlation between APOC1 and immune infiltrating cells in ESCA through STRING (https://cn.string-db.org/), the TISIDB (http://cis.hku.hk/TISIDB/) website, and various other analysis tools.

**Results:** In patients with ESCA, APOC1 was significantly more highly expressed in tumor tissues than in normal tissues (*p* < 0.001). APOC1 could diagnose ESCA more accurately and determine the TNM stage and disease classification with high accuracy (area under the curve, AUC≥0.807). The results of the Kaplan–Meier curve analysis showed that APOC1 has prognostic value for esophageal squamous carcinoma (ESCC) (*p* = 0.043). Univariate analysis showed that high APOC1 expression in ESCC was significantly associated with worse overall survival (OS) (*p* = 0.043), and multivariate analysis shows that high APOC1 expression was an independent risk factor for the OS of patients with ESCC (*p* = 0.030). In addition, the GO (gene ontology)/KEGG (Kyoto encyclopedia of genes and genomes) analysis showed a concentration of gene enrichment in the regulation of T-cell activation, cornification, cytolysis, external side of the plasma membrane, MHC protein complex, MHC class II protein complex, serine-type peptidase activity, serine-type endopeptidase activity, *Staphylococcus aureus* infection, antigen processing and presentation, and graft-versus-host disease (all *p* < 0.001). GSEA (gene set enrichment analysis) showed that enrichment pathways such as immunoregulatory-interactions between a lymphoid and non-lymphoid cell (NES = 1.493, p. adj = 0.023, FDR = 0.017) and FCERI-mediated NF-KB activation (NES = 1.437, p. adj = 0.023, FDR = 0.017) were significantly enriched in APOC1-related phenotypes. In addition, APOC1 was significantly associated with tumor immune infiltrating cells and immune chemokines.

**Conclusion:** APOC1 can be used as a prognostic biomarker for esophageal cancer. Furthermore, as a novel prognostic marker for patients with ESCC, it may have potential value for further investigation regarding the diagnosis and treatment of this group of patients.

## Introduction

Esophageal carcinoma (ESCA) currently ranks seventh and sixth in the world in terms of incidence and overall mortality, respectively (1). The main tissue types of ESCA are esophageal squamous cell carcinoma (ESCC) and esophageal adenocarcinoma (EAC) (2). Worldwide, ESCC accounts for approximately 90% of all patients with ESCA (3). East Asian countries including China have a high incidence of ESCA, and the dominant histological type is ESCC (4). Although multiple treatment modalities exist for patients with ESCA, the overall survival (OS) rate of patients remains poor, with a low (20%) 5-year survival rate (5, 6). Owing to the disease characteristics of ESCA itself and the complexity of the causative factors (7), failure to diagnose early lesions in time also has an important impact on the prognosis of patients (8).

In recent years, immunotherapy has been applied to the treatment of lung cancer, breast cancer, and other tumors with variable efficiency (9), and it is also highly anticipated that immunotherapy will benefit more patients with more tumor types (10). The safety of immunotherapy combined with radiotherapy in the treatment of ESCA is also under study (11). However, some patients do not respond to immunotherapy because of the multiple immune evasion mechanisms of the tumor (12). Current research has focused on radiotherapy, chemotherapy, and combination radiotherapy and immunotherapy, but there are no specific targeted molecules for the treatment of this malignancy (13). Therefore, it is particularly important to identify a biomarker that has high diagnostic sensitivity for ESCA and prognostic predictive value, and one that can be used to clarify the molecular mechanism of tumors and therapeutic targets.

APOC1 is a member of the apolipoprotein C family, with a size of 6.6 kD, and its coding gene region is located at 19q13.32 on chromosome 19 and is mainly synthesized by the liver (14). In recent years, several studies on the prognostic value of APOC1 in a variety of tumors have emerged (15-17). Some studies have shown that APOC1 regulates the development of various tumors such as renal cell carcinoma, colorectal cancer, and pancreatic cancer (18-21), which in turn affects the disease progression of many tumors.

It is well known that there is a strong link between inflammation and tumors (22), and infiltration of inflammatory cells is very common in tumor histopathology (23). The growth of tumors relies on the support of a complex mixture of organismal stroma containing inflammatory cells (24), and inflammatory indicators can predict the risk of postoperative tumor complications (25). Therefore, we hypothesized that the study of inflammation-related cells and proteins in the body could be used to predict tumor development. Studies have shown that several inflammation-related protein molecules including APOC1 are involved in the inflammatory response (26). Related studies have also shown that APOC1 may influence tumor progression in patients with kidney cancer (19) and colon cancer (18) by regulating gene expression and may be a potential prognostic marker for tumors such as gastric cancer (15) and lung cancer (17). However, to our knowledge, the expression and biological role of APOC1 in ESCA has not yet been reported except the study by Guo, Q et al. (27).

In this study, we applied several databases such as The Cancer Genome Atlas (TCGA), Gene Expression Omnibus (GEO), and the Genotype-Tissue Expression (GETx) (28-30); bioinformatics algorithms; and web tools such as the Search Tool for the Retrieval of Interacting Genes/Proteins (STRING) (31) and TISIDB(an integrated repository portal for tumor-immune system interactions) (32) to analyze the correlation between APOC1 and the tumor microenvironment of ESCA and to clarify the diagnostic and prognostic value, tumorigenic mechanism, and immune response regulation of APOC1 in ESCA.

At the beginning of this study, we analyzed the relationship between the expression of APOC1 in various tumors and disease prognosis from TCGA and GEO databases and specifically analyzed APOC1 expression in ESCA. Then, the diagnostic and prognostic value of APOC1 in esophageal cancer was evaluated, and finally, the relationship between APOC1 and tumor immune infiltrating cells was analyzed. With these results, we may be able to better understand the biological role of APOC1 in tumorigenesis and immunotherapeutic response in ESCA and ESCC.

## Materials and methods

### Data sources

We simultaneously extracted APOC1 expression and clinical data from the TCGA (https://portal.gdc.cancer.gov/) and GTEx (https://gtexportal.org/) databases in ESCA tissues, adjacent tumor tissues, and normal tissues to analyze the differences in APOC1 expression between tumor and normal tissues and the relationship between the expression of APOC1 and clinical characteristics. The data sets GSE161533 (84 paired normal tissues, para-tumor tissues, and tumor tissues from 28 ESCC patients microarray experiments performed by microarray analysis), GSE20347 (RNA was extracted from 17 paired tumor tissues and matched normal adjacent tissue from ESCC patients using the PureLink Micro-to-Midi Total RNA Purification System), and GSE29001(Gene expression from twelve cases of patient-matched normal basal epithelial cells, normal differentiated squamous epithelium, and cancer by expression microarrays.) were downloaded from the GEO (https://www.ncbi.nlm.nih.gov/gds/) database for the analysis of APOC1 expression differences between tumor and normal tissues and between tumor and adjacent tissues, respectively.

### Analysis of receiver operating characteristic (ROC) curve

After analyzing the difference between APOC1 expression in ESCA tissues and normal tissues, we performed a ROC curve analysis to detect the diagnostic ability of APOC1 for ESCA.

### Analysis of OS

Following the analysis of the diagnostic value of APOC1 for ESCA, we used Kaplan–Meier (KM) curves to analyze the influence of APOC1 expression status on the prognosis of patients with ESCA.

### Analysis of univariate and multivariate logistic regression

We compared the relationship between APOC1 expression status and the overall prognosis of ESCA patients by univariate and multifactorial analyses. We also analyzed whether APOC1 expression is an independent risk factor for the poor prognosis of ESCC patients. The significant criterion for COX analysis was set at *p* < 0.05.

### Analysis of functional enrichment

Differential analysis of APOC1 expression data (|log2 (fold change|) ≥1, adj. *p* < 0.05) was performed using the R (version 3.6.3) (R Core Team (2021). R: A language and environment for statistical computing. R Foundation for Statistical Computing, Vienna, Austria. URL https://www.R-project.org/) limma package. Gene Ontology/Kyoto Encyclopedia of Genes and Genome (GO/KEGG) analysis was used to obtain the correlation between APOC1 and ESCC, (adj. *p* < 0.05 was considered statistically significant), and the above results were visualized by applying the ggplot2 package (https://CRAN.R-project.org/package=ggplot2).

### Analysis of gene enrichment

Gene Set Enrichment Analysis (GSEA) was performed using the R (version 3.6.3) cluster profiler and ggplot2 packages. Statistical analysis with |NES| ((normalized enrichment score))>1, *p* < 0.05, and FDR (false discovery rate) > 0.25 was considered to indicate statistically significant differences.

### Analysis of protein interactions

In the protein interaction analysis, we used STRING online tool (31) to study and analyze the protein-protein binding to APOC1 protein-protein interaction (PPI) with APOC1. The network was operated to obtain 50 proteins that bind to APOC1, and the genes encoding these 50 proteins were intersected with the differentially expressed genes associated with APOC1 in ESCA tissues. We then analyzed the correlation analysis of APOC1 with these intersecting genes separately using Spearman’s relationship analysis. Statistical significance was considered at *p* < 0.05.

### Analysis of APOC1 expression and immune infiltration

We used the TISIDB (32) (http://cis.hku.hk/TISIDB/browse.php?gene=APOC1) web tool and Gene Set Variation Analysis (GSVA) method to analyze the relationship between the expression of APOC1 and tumor-infiltrating lymphocytes (TILs). The correlation between APOC1 and immune checkpoint genes in ESCA tissues was also analyzed, and the link between APOC1 expression and migration of immune cells was analyzed by chemokine and chemokine receptor modules.

### Statistics

We performed statistical analysis using the R (version 3.6.3) and plotted receiver operating characteristic (ROC) curves. The correlation between APOC1 mRNA expression and clinical characteristics of ESCA patients was tested using a chi-square test.

## Results

### Clinical characteristics of patients with ESCA

We extracted clinical data from the TCGA-GTEx database for 173 patients (162 patients with tumors and 11 healthy controls). The clinical information is shown in Table 1.

**TABLE 1:** Clinical characteristics of esophageal carcinoma (ESCA) patients.

Characteristic	Low expression of APOC1	High expression of APOC1	*p*
N	81	81	
Age, n (%)			0.346
<=60	45 (27.8%)	38 (23.5%)	
>60	36 (22.2%)	43 (26.5%)	
Gender, n (%)			0.177
Female	15 (9.3%)	8 (4.9%)	
Male	66 (40.7%)	73 (45.1%)	
Tumor central location, n (%)			0.168
Distal	55 (34.2%)	58 (36%)	
Mid	24 (14.9%)	18 (11.2%)	
Proximal	1 (0.6%)	5 (3.1%)	
T stage, n (%)			0.012
T1	18 (12.4%)	9 (6.2%)	
T2	21 (14.5%)	16 (11%)	
T3	31 (21.4%)	46 (31.7%)	
T4	0 (0%)	4 (2.8%)	
N stage, n (%)			0.424
N0	37 (25.7%)	29 (20.1%)	
N1	29 (20.1%)	34 (23.6%)	
N2	3 (2.1%)	6 (4.2%)	
N3	2 (1.4%)	4 (2.8%)	
M stage, n (%)			1.000
M0	57 (44.2%)	64 (49.6%)	
M1	4 (3.1%)	4 (3.1%)	
Histological type, n (%)			0.157
Adenocarcinoma	35 (21.6%)	45 (27.8%)	
Squamous Cell Carcinoma	46 (28.4%)	36 (22.2%)	
Pathologic stage, n (%)			0.005
Stage I	13 (9.2%)	3 (2.1%)	
Stage II	36 (25.4%)	33 (23.2%)	
Stage III	16 (11.3%)	33 (23.2%)	
Stage IV	4 (2.8%)	4 (2.8%)	
BMI, n (%)			0.040
<=25	36 (23.5%)	48 (31.4%)	
>25	42 (27.5%)	27 (17.6%)	
Reflux history, n (%)			0.967
No	42 (30.9%)	42 (30.9%)	
Yes	25 (18.4%)	27 (19.9%)	
Barrett’s esophagus, n (%)			0.511
No	51 (38.6%)	55 (41.7%)	
Yes	15 (11.4%)	11 (8.3%)	
Alcohol history, n (%)			0.635
No	21 (13.2%)	25 (15.7%)	
Yes	58 (36.5%)	55 (34.6%)	
Age, mean ± SD	61.32 ± 11.84	63.16 ± 12.2	0.332
BMI, median (IQR)	25.49 (22.12, 28.73)	23.74 (20.87, 26.61)	0.027

### Expression of APOC1 in various tumors and ESCA

**FIGURE 1:**
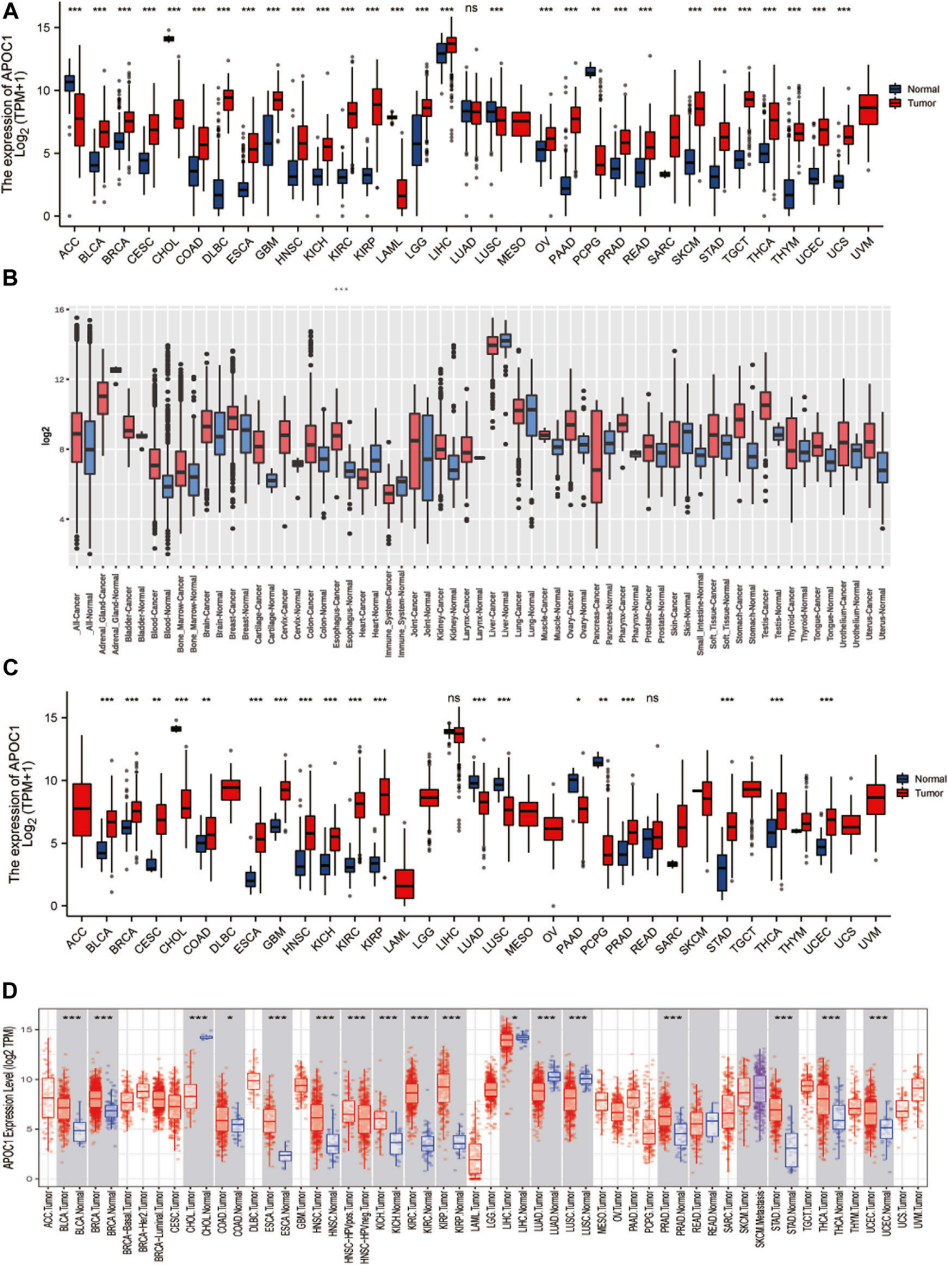
The expression of apolipoprotein C1 (APOC1) in various tumors including esophageal carcinoma (ESCA). Expression of APOC1 in tumor versus normal tissues based on the cancer genome atlas (TCGA) database from the genotype-tissue expression (GETx) **(A)** and the GEO platform 96 (GPL96) database from the gene expression database of normal and tumor tissues (GENT2) website **(B)**. The expression of APOC1 mRNA in tumor versus adjacent tissues based on the TCGA database from GTEx**(C)** and TCGA databases from the tumor immune estimation resource (TIMER) website**(D)**. *p < 0.05, **p < 0.01, ***p < 0.001.

**FIGURE 2:**
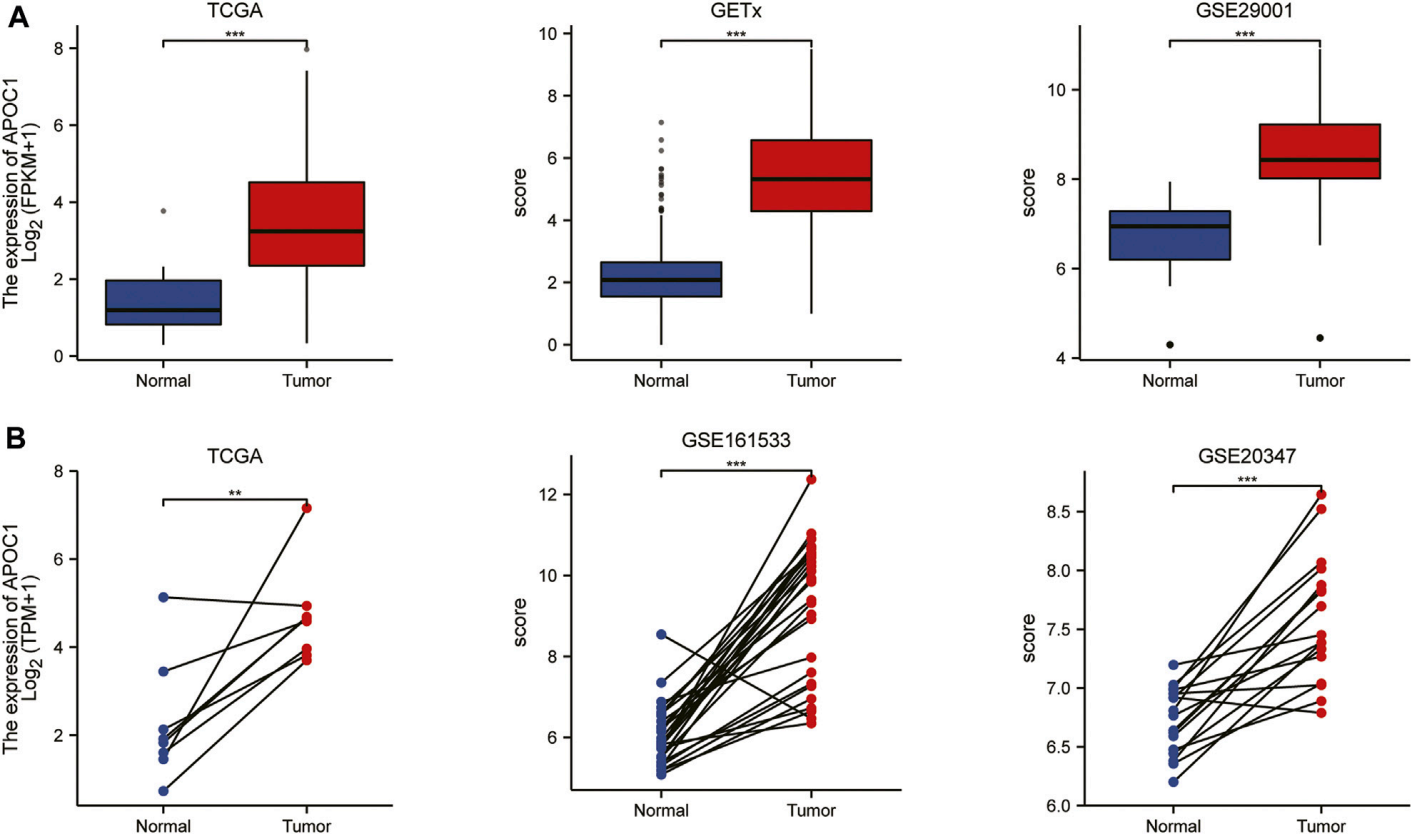
The expression of apolipoprotein C1 (APOC1) in esophageal carcinoma (ESCA) based on the cancer genome atlas (TCGA), Genotype-tissue expression (GTEx), and Gene expression omnibus (GEO) databases. The expression of APOC1 mRNA in ESCA and normal tissues **(A)**. The expression of APOC1 mRNA in ESCA and tumor adjacent tissues **(B)**. *p < 0.05, **p < 0.01, ***p < 0.001.

To understand the expression status of APOC1 in tumor tissues, adjacent tumor tissues, and normal tissues, we analyzed the expression of APOC1 in various types of tumors from the TCGA(10534 patients) and GTEx (15776 patients) databases. As shown in Figures 1A, B, the expression of APOC1 was significantly upregulated in tumor tissue compared with normal tissue in Breast invasive carcinoma (BRCA), Cervical squamous cell carcinoma and endocervical adenocarcinoma (CESC), Colon adenocarcinoma (COAD), ESCA, Kidney Chromophobe (KICH), Kidney renal clear cell carcinoma (KIRC), Kidney renal papillary cell carcinoma (KIRP), and Ovarian serous cystadenocarcinoma (OV). However, the expression level of APOC1 in tumors was significantly lower than in normal tissues in Adrenocortical carcinoma (ACC). APOC1 expression was not significantly different between Lung adenocarcinoma (LUAD) and normal tissues. We also analyzed APOC1 expression in various tumor tissues and tumor-adjacent tissues. APOC1 was more highly expressed in tumor tissues than tumor adjacent in Bladder Urothelial Carcinoma (BLCA), COAD, Head and Neck squamous cell carcinoma (HNSC), KICH, KIRC, KIRP, Prostate adenocarcinoma (PRAD), Stomach adenocarcinoma (STAD), Thyroid carcinoma (THCA), and Uterine Corpus Endometrial Carcinoma (UCEC), while it was less expressed in tumor tissues than tumor adjacent tissues in Cholangiocarcinoma (CHOL), LUAD, and LUSC(lung squamous cell carcinoma), with no significant difference in expression in reading (Figures 1C, D). Next, we analyzed the transcription levels of APOC1 with the TCGA, GTEx, and GEO databases. The analysis showed that APOC1 mRNA expression was significantly upregulated in ESCA tissues compared with normal tissues (*p* < 0.001) (Figure 2A), and the expression of APOC1 mRNA was also significantly increased in ESCA tissues compared with tumor adjacent tissues (*p* < 0.01) (Figure 2B).

### Diagnostic value of APOC1 mRNA in ESCA

The diagnostic value of APOC1 mRNA expression in ESCA was analyzed in our study using the ROC method. Our results showed that the area under the curve (AUC) was 0.887. We also analyzed the diagnostic value of APOC1 mRNA expression in different stages, grading, lymph node metastasis, and distant metastasis. The AUC values were 0.808, 0.895, 0.926, and 0.898 by stage I, stage II, stage III, and stage IV, respectively; 0.862, 0.899, 0.900, and 1.000 by T stage T1, T2, T3, and T4, respectively; 0.884, 0.890, 0.949, and 0.955 by lymph node metastasis stages N0, N1, N2, and N3, respectively; and 0.898 for both M0 and M1 according to M staging (Supplementary Figure S1).

### Expression of APOC1 is an independent risk factor for poor outcomes in patients with ESCC

**FIGURE 3:**
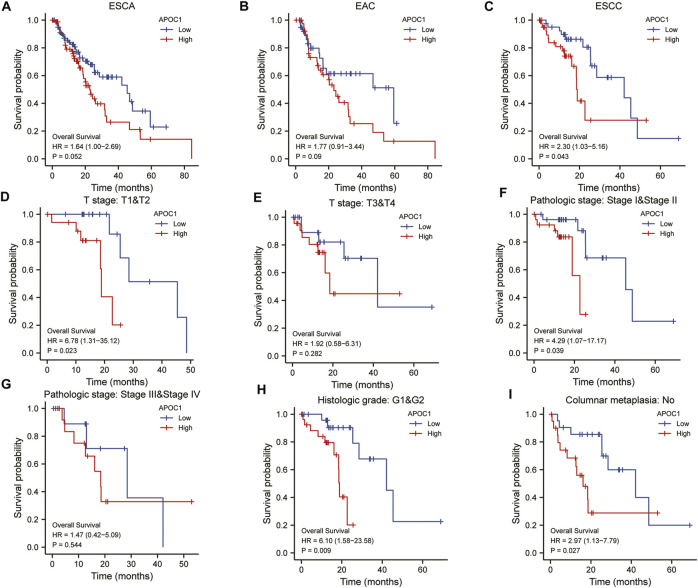
Analysis of overall survival (OS) with apolipoprotein C1 (APOC1) mRNA expression in esophageal carcinoma (ESCA), esophageal adenocarcinoma (EAC), and esophageal squamous cell carcinoma (ESCC) **(A–C)**. Subgroup analysis of overall survival with APOC1 mRNA in ESCC **(D–I)**.

**FIGURE 4:**
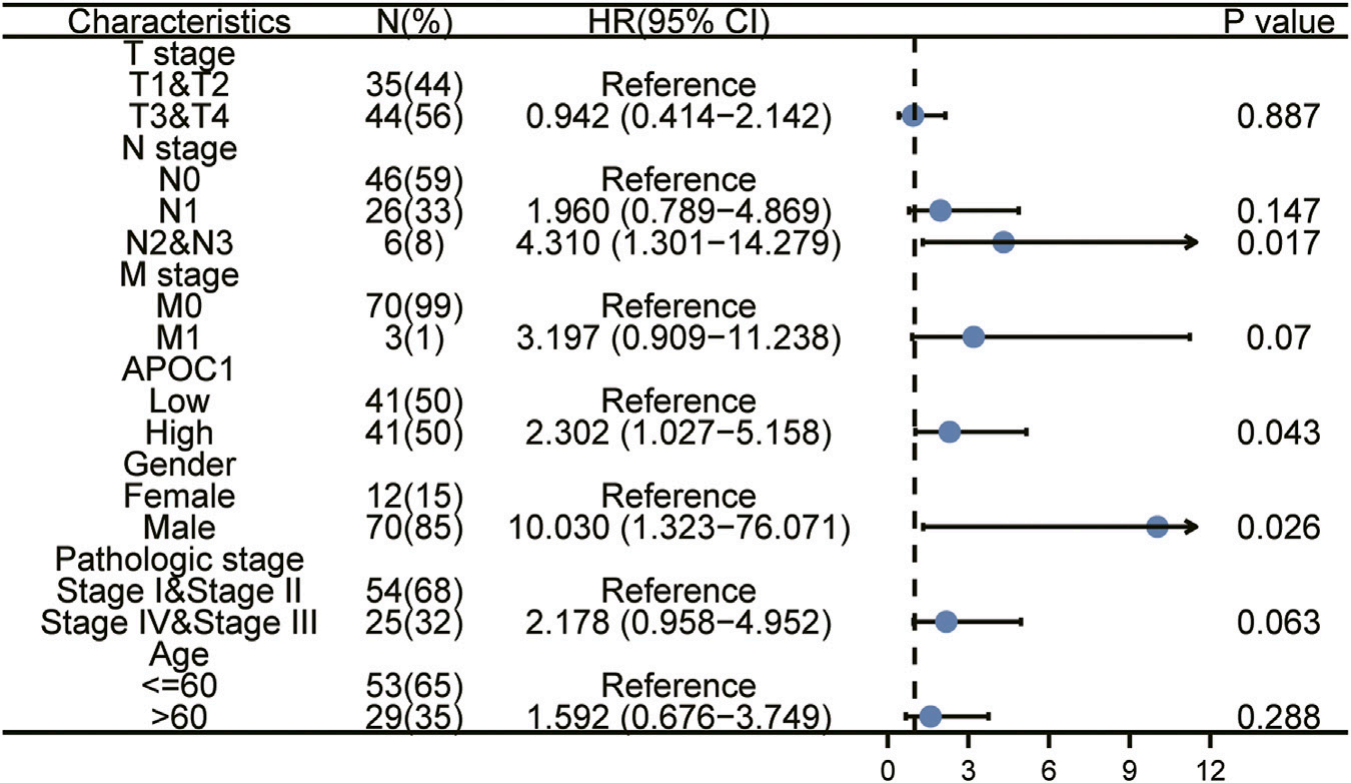
Univariate and multivariate regression analysis of apolipoprotein C1 (APOC1) and characteristics with overall survival (OS) in ESCC patients.

We applied the K-M survival curve to analyze the prognostic impact of APOC1 mRNA expression on patients with ESCA. The results showed that APOC1 mRNA expression had no significant effect on all tumors including EAC (*p* = 0.052) (Figure 3A) and EAC alone (*p* = 0.09) (Figure 3B), while patients with ESCC who overexpressed APOC1 mRNA in their tissues had a worse survival prognosis (*p* = 0.043) (Figure 3C). Overexpression of APOC1 mRNA also significantly affected the OS of patients with ESCC at T-stage (T1 and T2) (*p* = 0.023) (Figure 3D), pathological stage (TNM-stage, AJCC prognostic stage) (stage I and stage II) (*p* = 0.039) (Figure 3E), tumor histological grade (grade 1 and grade 2) (*p* = 0.009) (Figure 3F), and columnar metaplasia (*p* = 0.027) ESCC (Figure 3G), while the effect was not significant in T-stage (T3 and T4) (*p* = 0.282) (Figure 3H) and pathological stage (TNM-stage, AJCC prognostic stage) (Stage III and Stage IV) (*p* = 0.544) (Figure 3I) as shown in the subgroup analysis. Univariate and multifactorial analyses showed that high expression of APOC1mRNA, lymph node metastasis, and sex were associated with OS of patients with ESCC, and APOC1 mRNA was likely an independent prognostic risk factor for OS in patients with ESCC (Table 2; Figure 4).

**TABLE 2:** Cox proportional hazards regression model analysis for OS of ESCC patients.

Characteristics	Total (N)	Univariate analysis		Multivariate analysis	
		Hazard ratio (95% CI)	*p* value	Hazard ratio (95% CI)	*p* value
T stage	79				
T1&T2	35	Reference			
T3&T4	44	0.942 (0.414–2.142)	0.887		
N stage	78				
N0	46	Reference			
N1	26	1.960 (0.789–4.869)	0.147	1.315 (0.353–4.896)	0.684
N2&N3	6	4.310 (1.301–14.279)	**0.017**	7.977 (1.200–53.005)	**0.032**
M stage	73				
M0	70	Reference			
M1	3	3.197 (0.909–11.238)	0.070	4.643 (0.878–24.553)	0.071
APOC1	82				
Low	41	Reference			
High	41	2.302 (1.027–5.158)	**0.043**	3.210 (1.119–9.208)	**0.030**
Gender	82				
Female	12	Reference			
Male	70	10.030 (1.323–76.071)	**0.026**	191838562.424 (0.000–Inf)	0.998
Pathologic stage	79				
Stage I&Stage II	54	Reference			
Stage IV&Stage III	25	2.178 (0.958–4.952)	0.063	0.512 (0.123–2.133)	0.358
Age	82				
<=60	53	Reference			
>60	29	1.592 (0.676–3.749)	0.288		

### Functional analysis of APOC1 in ESCC

**FIGURE 5:**
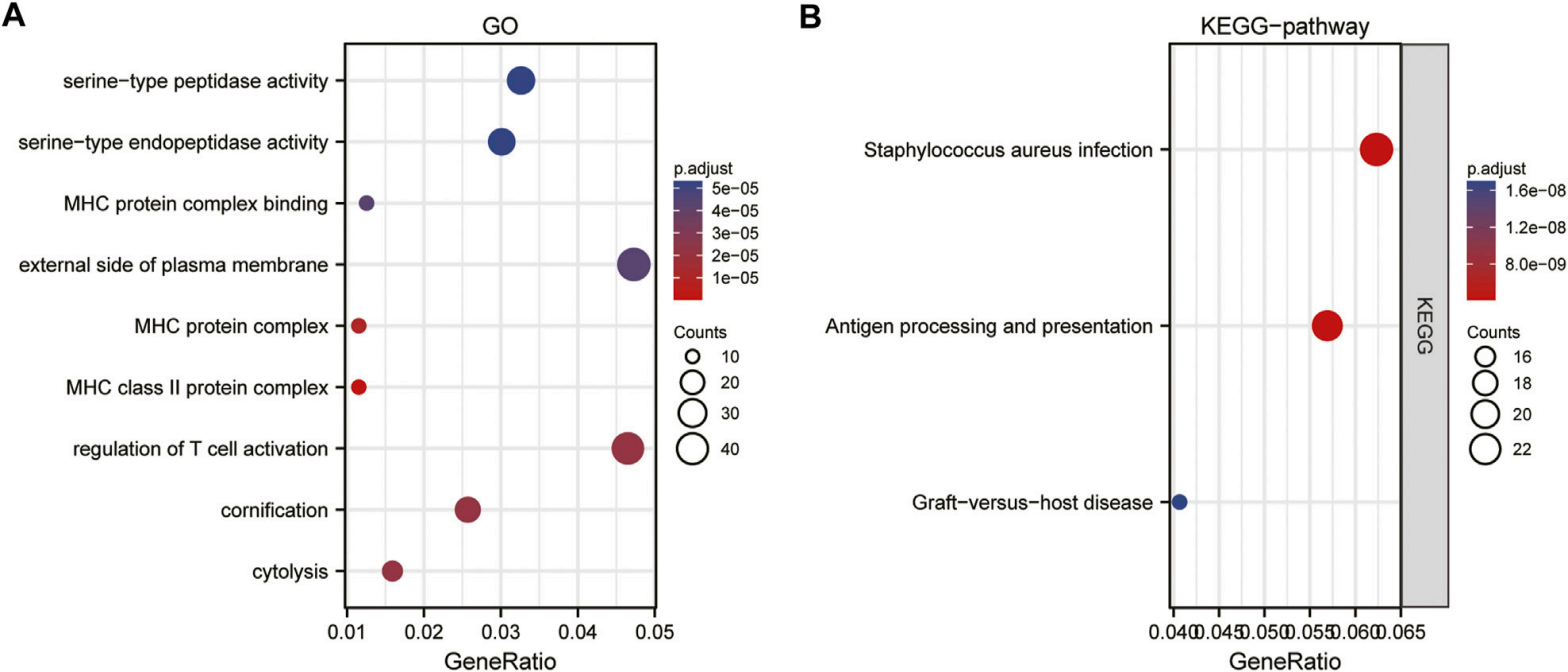
Gene ontology (GO)(**A)** and kyoto encyclopedia of genes and genome (KEGG) **(B)**enrichment analysis of apolipoprotein C1 (APOC1) expression correlated different expression genes in esophageal squamous cell carcinoma (ESCC).

GO functional annotation analysis revealed that APOC1 co-expressed genes in ESCC were mainly involved in the following aspects: regulation of T cell activation, cornification, cytolysis, external side of plasma membrane, MHC protein complex, MHC class II protein complex, serine−type peptidase activity, serine-type endopeptidase activity (Supplementary Table S1; Figure 5A). The KEGG pathway enrichment analysis showed that co-expressed genes of APOC1 were mainly enriched in *Staphylococcus aureus* infection, antigen processing and presentation, and graft-versus-host disease pathways (Supplementary Table S2; Figure 5B).

### Identification of APOC1-related signaling pathways by GSEA analysis

**FIGURE 6:**
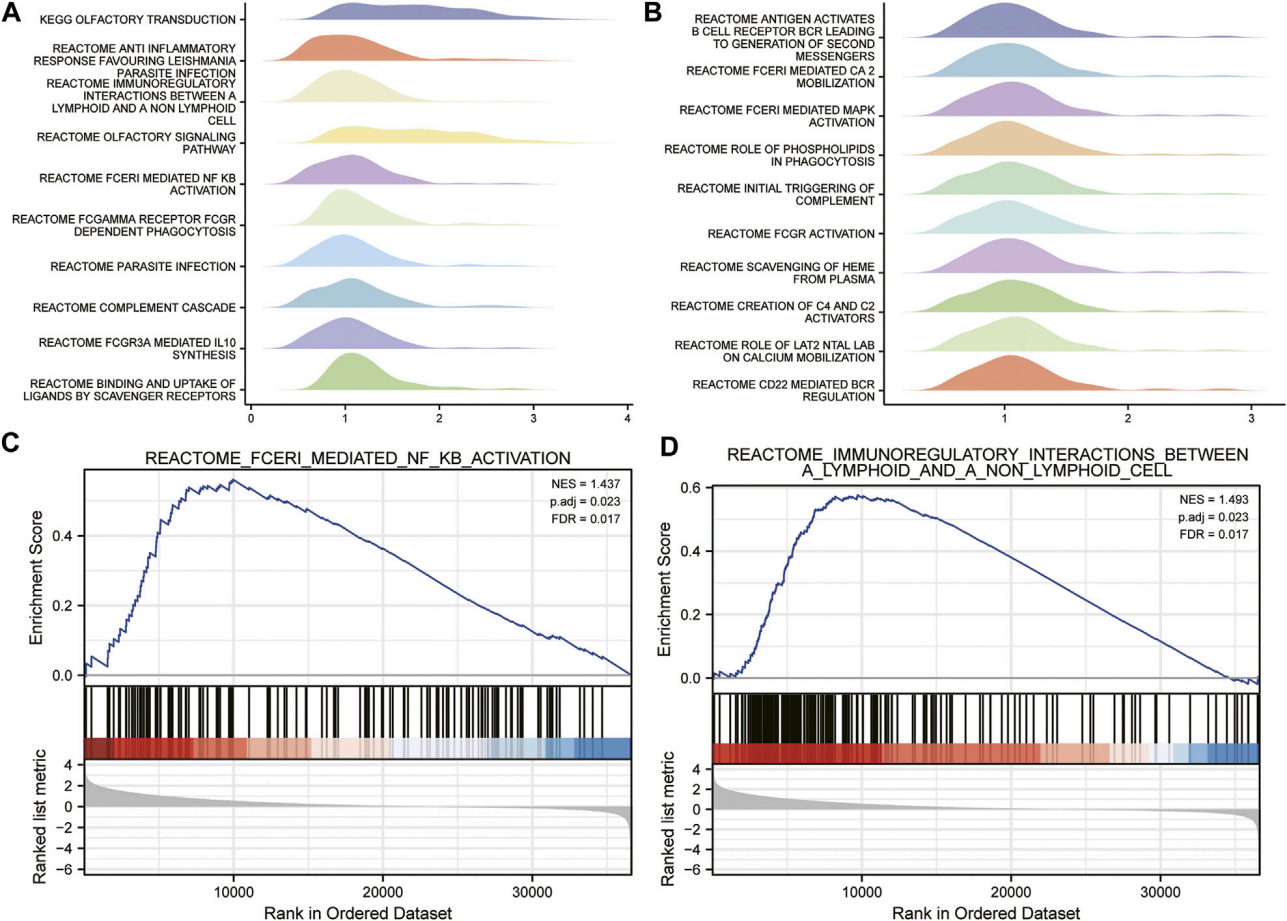
Identification of apolipoprotein C1 (APOC1) related signaling pathways by gene set enrichment analysis (GSEA) analysis **(A,B)**. Fceri mediated nuclear factor kappa B (NFKB) activation **(C)**. Immuno-regulatory interactions between a lymphoid and a non-lymphoid cell **(D)**.

The signaling pathways associated with ESCC were analyzed by the GSEA method. The results showed that immunoregulatory interactions between a lymphoid and a non-lymphoid cell and FCERI-mediated NF-KB activation were significantly enriched in APOC1-related phenotypes were significantly enriched (Figure 6).

### Establishment of protein interactions signaling network

**FIGURE 7:**
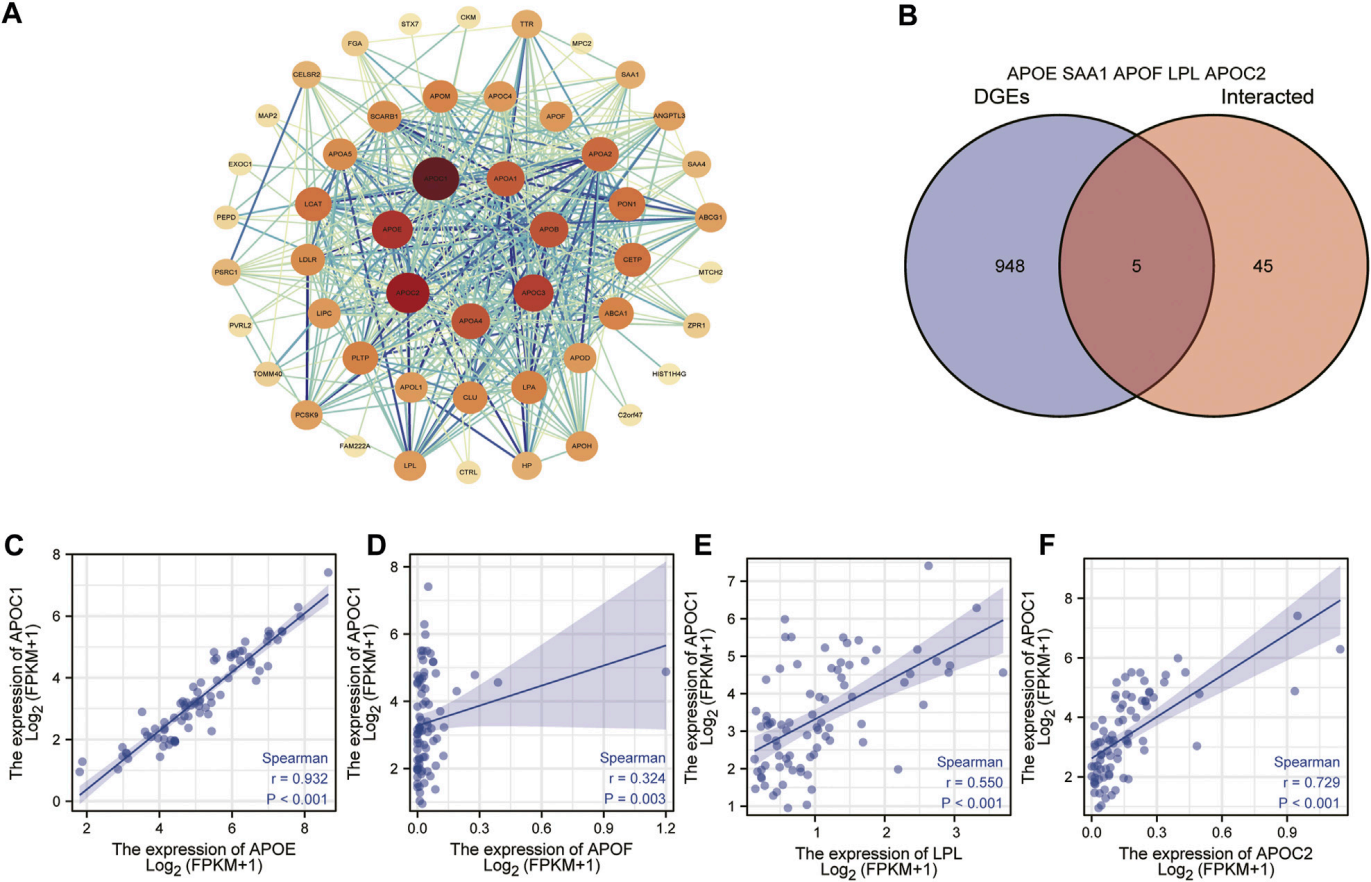
Establishment of apolipoprotein C1 (APOC1) related genes protein interactions signaling network. Protein-protein interaction (PPI) network obtained from the search tool for the retrieval of interacting genes/proteins (STRING) **(A)**. Common proteins screened by taking intersections between APOC1-interacting proteins and APOC1 expression-related differential gene proteins **(B)**. Correlation of APOC1 with common proteins **(C–F)**.

Through the establishment of protein interactions signaling network, we can understand the metabolic and molecular mechanism of ESCC. Therefore, we analyzed the interaction of APOC1 in ESCC through the STRING website protein PPI network. First, on the STING website, we set the parameters for text mining and experimental evidence identification, and then we obtained the APOC1-related protein interactions network (Figure 7A). In addition, we also took the proteins interacting with APOC1 and the differential proteins associated with APOC1 expression in ESCC for intersection analysis and screened the common proteins as APOE, SAA1, APOF, LPL, APOC2, respectively (Figure 7B). Next, we analyzed the correlation between the expression of APOC1 and that of these common proteins, and the results showed that APOC1 had a significant positive correlation with the expression of APOE (r = 0.324, *p* = 0.003); APOF (r = 0.932, *p* < 0.001); LPL (r = 0.550, *p* < 0.001); and APOC2 (r = 0.729, *p* < 0.001) (Figures 7C–F).

### Correlation between the expression of APOC1 and the level of immune infiltration

**FIGURE 8:**
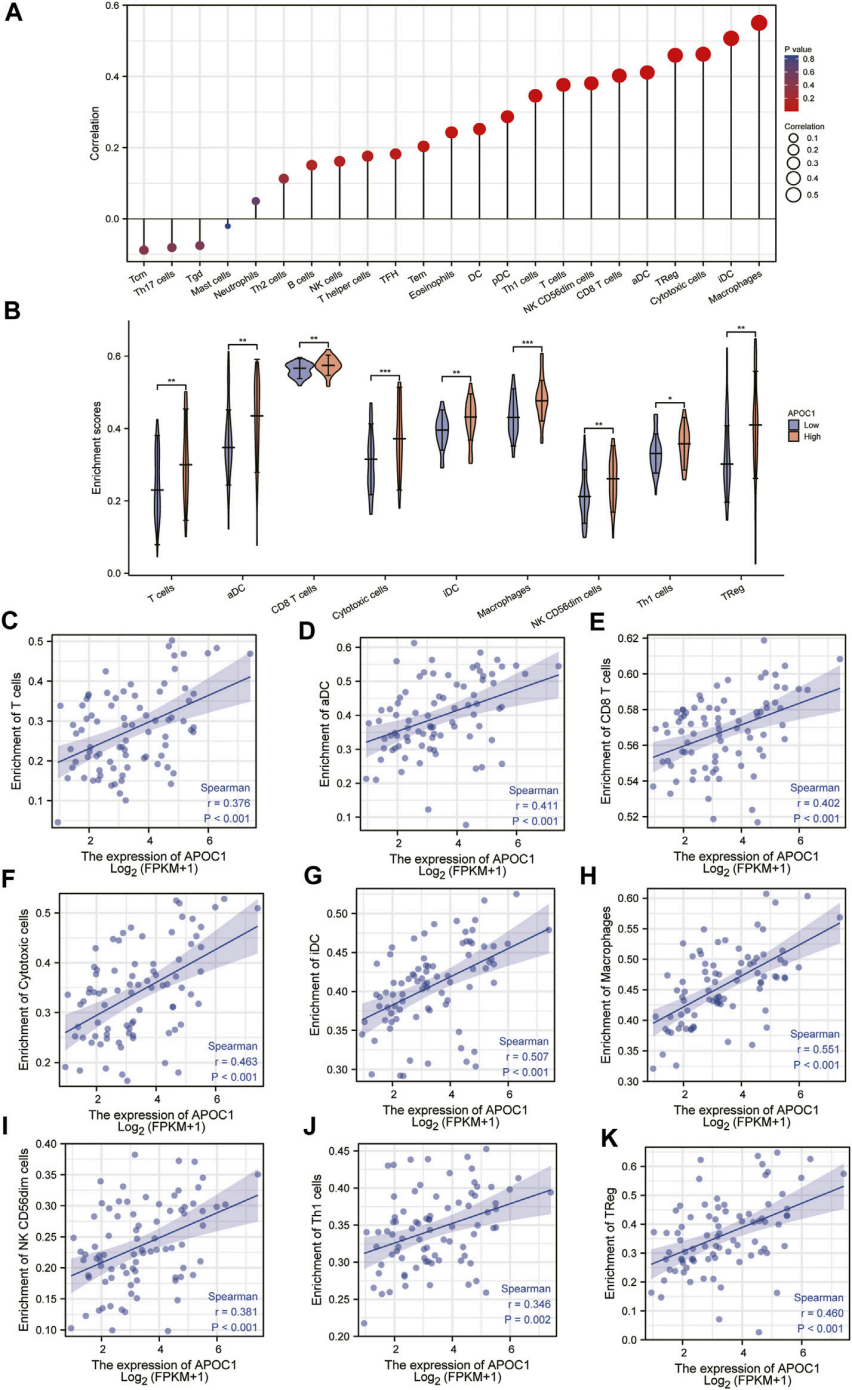
Correlation between the expression of apolipoprotein C1 (APOC1) and immune infiltration level in esophageal squamous cell carcinoma (ESCC) **(A,B)**. Positive correlation between APOC1 and various immune infiltrating cells **(C–K)**.

**FIGURE 9:**
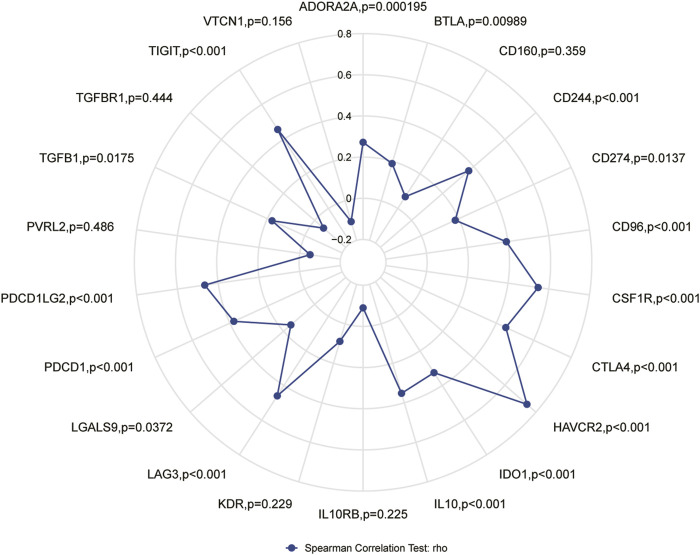
Correlation of apolipoprotein C1 (APOC1) expression with immune checkpoint genes.

The results of this study showed that in ESCC tissue, a variety of immune infiltrating cells were positively correlated with APOC1 expression such as T cells (r = 0.376, *p* < 0.001); aDC (antibody-dependent cytotoxicity) (r = 0.411, *p* < 0.001); CD8T cells (r = 0.402, *p* < 0.001), cytotoxic cells (r = 0.463, *p* < 0.001), iDC (immature dendritic cell) (r = 0.507, *p* < 0.001), macrophages (r = 0.551, *p* < 0.001), NK CD56 dim cells (r = 0.381, *p* < 0.001), Th1 cells (r = 0.346, *p* = 0.002), and Treg (r = 0.460, *p* < 0.001) (Figures 8A, B). Moreover, cell grouping analysis and individual cell analysis showed a significant positive correlation between APOC1 and immune infiltrating cells (Figures 8C–K). To further explore the relationship between APOC1 expression and tumor-infiltrating lymphocytes (TILs), we examined the relationship between TILs and APOC1 expression through the TISIDB website. The results showed a significant positive correlation between APOC1 and multiple TILs cells in ESCA (Supplementary Figure S2). Given that current targeted drug therapy against tumors benefits patients with a wide range of tumors (33), in this study we also analyzed the relationship between APOC1 and various tumor control-related genes. Our results showed that tumor targeting-related immune checkpoint genes such as CD274 (*p* = 0.0137), TGFB1 (*p* = 0.0175), PDCD1 (*p* < 0.001), CTLA4 (*p* < 0.001), and LAG3 (*p* < 0.001) were significantly associated with APOC1 (Figure 9). These significant correlations may be related to the poorer prognosis of patients with ESCC owing to high expression of APOC1. Interestingly, APOC1 expression was also significantly correlated with inflammatory factors such as IL10 (*p* < 0.001) (Figure 9). In addition, we also analyzed the relationship between APOC1 expression and chemokines (Supplementary Figure S3) and chemokine receptor (Supplementary Figure S4). The analysis showed a significant positive correlation between APOC1 and a large variety of immune chemokines and chemokine receptors. Combined with the results of correlation analysis of APOC1 expression with target molecules, immune-related molecules, and inflammatory chemokines, AOPC1 may be involved in immune infiltration and inflammatory responses in the tumor microenvironment and tumorigenesis and development.

## Discussion

In the present study, we found that consistent with the findings on the role of APOC1 in other cancers, APOC1 also showed significantly higher expression in ESCA tissues than in normal tissues and tumor-adjacent tissues. Therefore, we hypothesized that APOC1 may play an important role in the development of ESCA. Subsequently, we found by ROC analysis that the expression of APOC1 in different patient stages of ESCA also has some diagnostic value. In subsequent survival analysis, it was shown that high APOC1 expression was significantly associated with poorer OS in patients with ESCC. Based on these findings, we concluded that APOC1 could be used as a biomarker for the staging determination and prognosis of patients with ESCA, especially ESCC.

Tumorigenesis and the development of tumors depend on the tumor microenvironment (TME), and tumors are also involved in the composition, regulation, and development of the TME (34). The effectiveness of tumor immunotherapy is related not only to the tumor, but also to the immune microenvironment, which is external to the tumor (35). By contrast, tumor-infiltrating lymphocytes with a predictive role for immune checkpoint inhibitors in ESCA immunotherapy have not yet been reported. In the present study, we analyzed the correlation of APOC1 expression with immune checkpoint markers and tumor immune infiltrating lymphocytes. Our results showed that APOC1 expression was significantly and positively correlated with aDC (r = 0.411, *p* < 0.001); CD8 T cells (r = 0.402, *p* < 0.001); and cytotoxic cells (r = 0.463, *p* < 0.001), especially macrophages (r = 0.551, *p* < 0.001). Further analysis showed that APOC1 had significant positive correlations with IL10 (*p* < 0.001), CSF1R (*p* < 0.001), CD274 (PD-L1) (*p* = 0.0137), TGFB1 (*p* = 0.0175), PDCD1 (PD-1) (*p* < 0.001), PDCD1LG2 (PD-L2) (*p* < 0.001), CTLA4(*p* < 0.001), and LAG3(*p* < 0.001). This may be because the TME with high APOC1 expression affects the polarization of macrophages, causing macrophages to secrete substances such as IL10, which in turn causes pro-tumorigenic effects of macrophages on ESCA cells, leading to tumor development (36). It was inferred that APOC1 expression was significantly correlated with multiple immune markers and immune infiltration levels, which is consistent with previous findings (37). Because APOC1 has a close relationship with both tumor internal factors and tumor external factors of the tumor immune microenvironment (TIME), we hypothesized that the expression level of APOC1 may be a potential predictive biomarker for application as an immune checkpoint inhibitor. According to the results of GSEA analysis, immunoregulatory interactions between a lymphoid and a non-lymphoid cell and FCERI-mediated NF-ΚB activation are potential signaling pathways, suggesting that APOC1 may be closely related to both cellular and humoral immune responses during esophageal carcinogenesis. This may also be a potential mechanism for the association of APOC1 with poorer prognosis in patients with ESCA. Meanwhile, the analysis of the relationship between APOC1 expression and related immune chemokines showed that APOC1 was significantly and positively correlated with most of the immune chemokines, and chemokines regulate lymphocyte recruitment(38), which may be associated with poorer prognosis of ESCA. However, thus far to our knowledge, no experiments have been conducted to verify this inference, which is a limitation of our study. Further basic and clinical experiments are needed to fully elucidate the biological function of APOC1 in ESCA.

## Conclusion

In conclusion, APOC1 may be used as a diagnostic biomarker for esophageal cancer. In addition, as a new prognostic marker in ESCC patients, it positively correlates with T cells, CD8T cells, cytotoxic cells and is closely related to NF-KB, and may be potentially valuable for further investigation of the diagnosis and treatment of ESCC patients.

## Data Availability

The original contributions presented in the study are included in the article/Supplementary Material, further inquiries can be directed to the corresponding authors.

## Abbreviations

APOC1, Apolipoprotein C1; AUC, area under the curve; ACC, adrenocortical carcinoma; BLCA, bladder urothelial carcinoma; BRCA, Breast invasive carcinoma; CESC, endocervical adenocarcinoma; CHOL, paraneoplastic tissues in cholangiocarcinoma; COAD, Colon adenocarcinoma; EAC, esophageal adenocarcinoma; ESCC, esophageal squamous cell carcinoma; GEO, Gene Expression Omnibus; GO, gene ontology; GSEA, gene set enrichment analysis; GSVA, gene set variation analysis; HNSC, neck squamous cell carcinoma; KEGG, kyoto encyclopedia of genes and genomes; KIRC, kidney renal clear cell carcinoma; KICH, kidney chromophobe; KIRP, kidney renal papillary cell carcinoma; LUAD, lung adenocarcinoma; LUSC, lung squamous cell carcinoma; OS, overall survival; OV, ovarian serous cystadenocarcinoma; PRAD, prostate adenocarcinoma; STAD, stomach adenocarcinoma; TCGA, the cancer genome atlas; THCA, thyroid carcinoma; TILs, tumor-infiltrating lymphocytes; UCEC, uterine corpus endometrial carcinoma.
