# Supplementary material for: APOC1 predicts a worse prognosis for esophageal squamous cell carcinoma and is associated with tumor immune infiltration during tumorigenesis
Source: Pathol Oncol Res. 2023 Mar 8;29:1610976. doi: 10.3389/pore.2023.1610976 (PMC10030600; doi:10.3389/pore.2023.1610976)
Supplement: Supplementary file 1 [file Table1.docx]

| Table S1 GO ¬enrichment analysis of APOC1 expression correlated different expression genes in ESCC. | | | | | | | |
| --- | --- | --- | --- | --- | --- | --- | --- |
| Ontology | ID | Description | GeneRatio | BgRatio | pvalue | p.adjust | qvalue |
| BP | GO:0019835 | cytolysis | 13/817 | 40/18670 | 7.88e-09 | 2.21e-05 | 1.97e-05 |
| BP | GO:0050863 | regulation of T cell activation | 38/817 | 314/18670 | 1.42e-08 | 2.21e-05 | 1.97e-05 |
| BP | GO:0070268 | cornification | 21/817 | 112/18670 | 1.48e-08 | 2.21e-05 | 1.97e-05 |
| CC | GO:0042613 | MHC class II protein complex | 10/867 | 16/19717 | 1.62e-10 | 7.39e-08 | 6.26e-08 |
| CC | GO:0042611 | MHC protein complex | 10/867 | 25/19717 | 4.60e-08 | 1.05e-05 | 8.90e-06 |
| CC | GO:0009897 | external side of plasma membrane | 41/867 | 393/19717 | 2.89e-07 | 4.40e-05 | 3.73e-05 |
| MF | GO:0023023 | MHC protein complex binding | 10/797 | 25/17697 | 5.73e-08 | 4.34e-05 | 3.74e-05 |
| MF | GO:0008236 | serine-type peptidase activity | 26/797 | 182/17697 | 1.82e-07 | 5.32e-05 | 4.59e-05 |
| MF | GO:0004252 | serine-type endopeptidase activity | 24/797 | 160/17697 | 2.13e-07 | 5.32e-05 | 4.59e-05 |
