# Supplementary figures and images for: APOC1 predicts a worse prognosis for esophageal squamous cell carcinoma and is associated with tumor immune infiltration during tumorigenesis
Source: Pathol Oncol Res. 2023 Mar 8;29:1610976. doi: 10.3389/pore.2023.1610976 (PMC10030600; doi:10.3389/pore.2023.1610976)

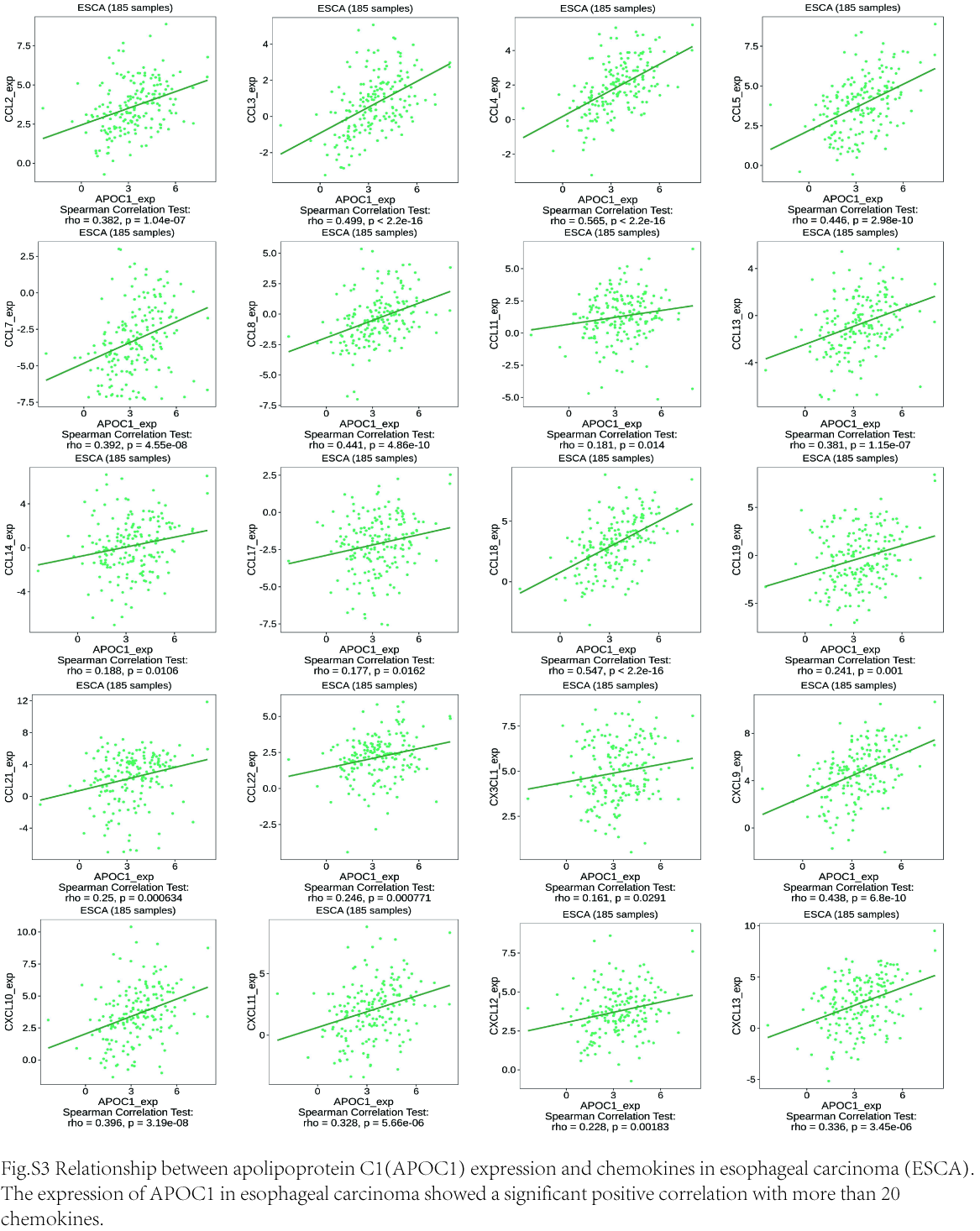

Supplement: Supplementary file 2 [file Image3.TIF]

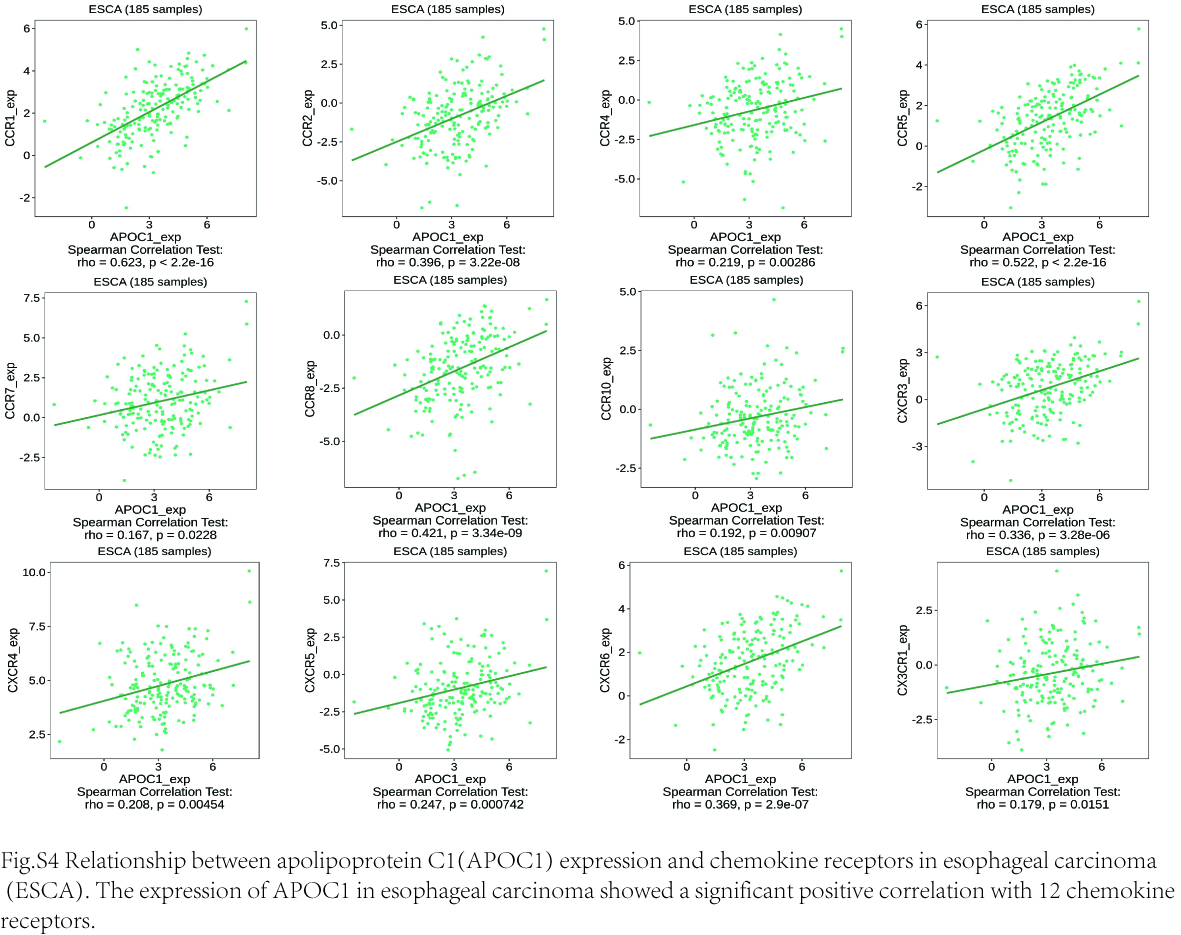

Supplement: Supplementary file 3 [file Image4.TIF]

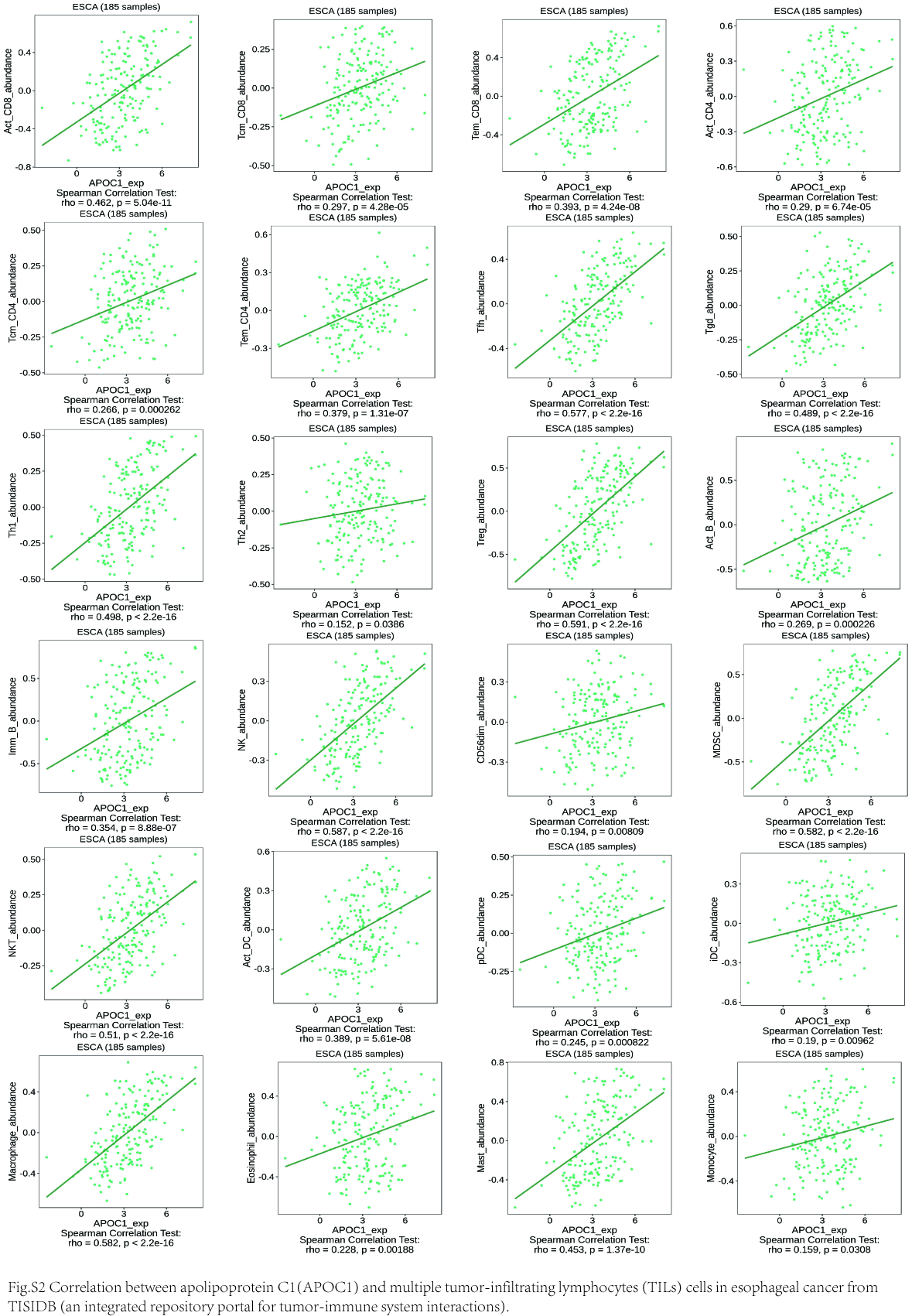

Supplement: Supplementary file 4 [file Image2.TIF]

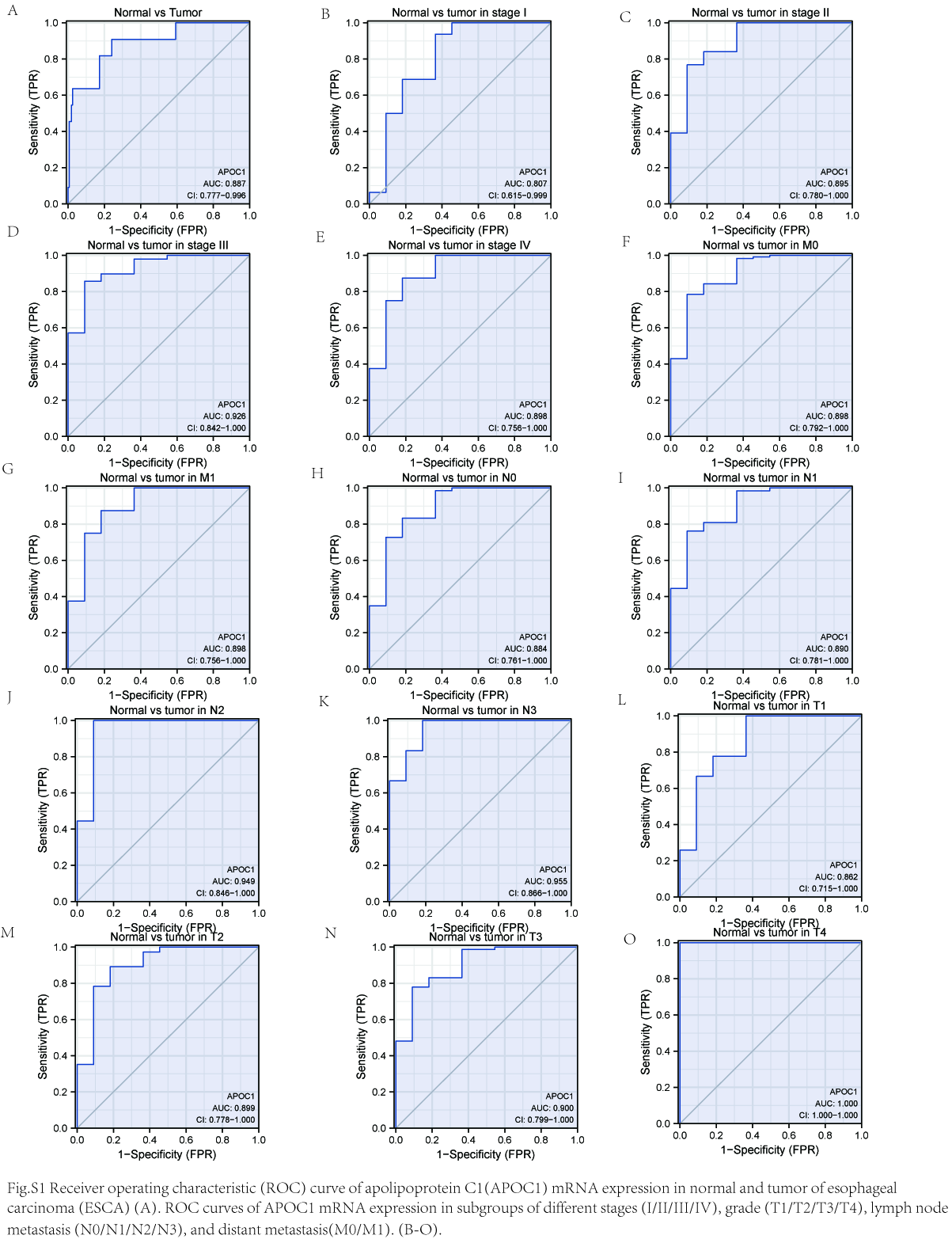

Supplement: Supplementary file 5 [file Image1.TIF]
