# Supplementary material for: APOC1 predicts a worse prognosis for esophageal squamous cell carcinoma and is associated with tumor immune infiltration during tumorigenesis
Source: Pathol Oncol Res. 2023 Mar 8;29:1610976. doi: 10.3389/pore.2023.1610976 (PMC10030600; doi:10.3389/pore.2023.1610976)
Supplement: Supplementary file 6 [file Table2.docx]

| Table S2 KEGG­ enrichment analysis of APOC1 expression correlated different expression genes in ESCC | | | | | | | |
| --- | --- | --- | --- | --- | --- | --- | --- |
| Ontology | ID | Description | GeneRatio | BgRatio | pvalue | p.adjust | qvalue |
| KEGG | hsa04612 | Antigen processing and presentation | 21/369 | 78/8076 | 2.04e-11 | 4.09e-09 | 3.53e-09 |
| KEGG | hsa05150 | Staphylococcus aureus infection | 23/369 | 96/8076 | 3.05e-11 | 4.09e-09 | 3.53e-09 |
| KEGG | hsa05332 | Graft-versus-host disease | 15/369 | 42/8076 | 1.90e-10 | 1.70e-08 | 1.47e-08 |
